# A polygenic score for age‐at‐first‐birth predicts disinhibition

**DOI:** 10.1111/jcpp.13224

**Published:** 2020-03-27

**Authors:** Leah S. Richmond‐Rakerd, Terrie E. Moffitt, Louise Arseneault, Daniel W. Belsky, Jennie Connor, David L. Corcoran, HonaLee Harrington, Renate M. Houts, Richie Poulton, Joey A. Prinz, Sandhya Ramrakha, Karen Sugden, Jasmin Wertz, Benjamin S. Williams, Avshalom Caspi

**Affiliations:** ^1^ Department of Psychology and Neuroscience Duke University Durham NC USA; ^2^ Frank Porter Graham Child Development Institute University of North Carolina at Chapel Hill Chapel Hill NC USA; ^3^ Department of Psychiatry and Behavioral Sciences Duke University School of Medicine Durham NC USA; ^4^ Center for Genomic and Computational Biology Duke University Durham NC USA; ^5^ Institute of Psychiatry, Psychology, and Neuroscience King's College London London UK; ^6^ Department of Epidemiology Columbia University Mailman School of Public Health New York NY USA; ^7^ Department of Preventive and Social Medicine University of Otago Dunedin New Zealand; ^8^ Dunedin Multidisciplinary Health and Development Research Unit Department of Psychology University of Otago Dunedin New Zealand

**Keywords:** Reproductive behavior, self‐control, risk‐taking, genetics, longitudinal

## Abstract

**Background:**

A recent genome‐wide association study identified molecular‐genetic associations with age‐at‐first‐birth. However, the meaning of these genetic discoveries is unclear. Drawing on evidence linking early pregnancy with disinhibitory behavior, we tested the hypothesis that genetic discoveries for age‐at‐first‐birth predict disinhibition.

**Methods:**

We included participants with genotype data from the two‐decade‐long Environmental Risk (E‐Risk) Study (*N* = 1,999) and the four‐decade‐long Dunedin Study (*N* = 918). We calculated a genome‐wide polygenic score for age‐at‐first‐birth and tested whether it was associated with a range of disinhibitory outcomes across the life course, including low childhood self‐control; risk for externalizing psychopathology; officially recorded criminal offending; substance dependence; informant reports of disinhibitory problems; and number of lifetime sexual partners. We further tested whether associations were attributable to accelerated pubertal maturation.

**Results:**

In both cohorts, the age‐at‐first‐birth polygenic score predicted low childhood self‐control, externalizing psychopathology, officially recorded criminal offending, substance dependence, and number of sexual partners. Associations were modest, but robust across replication. Childhood disinhibition partly mediated associations between the polygenic score and reproductive behaviors. In contrast, associations were not attributable to accelerated pubertal timing.

**Conclusions:**

Genomic discoveries for age‐at‐first‐birth are about more than reproductive biology: They provide insight into the disinhibitory traits and behaviors that accompany early parenthood. Age‐at‐first‐birth is a useful proxy phenotype for researchers interested in disinhibition. Further, interventions that improve self‐regulation abilities may benefit young parents and their children.

## Introduction

The birth of a first child is a salient life event, and there is marked variation in whether and when individuals meet this reproductive milestone. Until recent decades, research largely considered socioenvironmental explanations for variation in age‐at‐first‐birth and explanations of the changing prevalence of early pregnancy depended primarily on sociocultural factors (Balbo, Billari, & Mills, 2013). However, with the advent of fertility control, attention has turned to individual differences in early childbearing, shifting the question from ‘*how many* individuals have an early pregnancy?’ to ‘*who* has an early pregnancy?’ Some of these individual differences are attributable to genetic differences between people. Early childbearing is heritable (Harden, 2014), and a genome‐wide association study (GWAS) of 251,151 individuals identified molecular‐genetic associations with age‐at‐first‐birth (Barban et al., 2016). What remains unclear is what these molecular‐genetic discoveries mean. Do they capture biological underpinnings of fertility? Or, do they index psychological traits and behaviors that accompany early parenthood? We tested the hypothesis that genetic influences on age‐at‐first‐birth – as measured by a polygenic score – predict disinhibition.

We derived this hypothesis from five lines of study. First, early pregnancy often results from risky sexual behavior (Klein & Committee on Adolescence, 2005). Second, young parents exhibit other disinhibitory behaviors including antisocial behavior and substance misuse (Coyne & D'Onofrio, 2012). Third, low self‐control predicts early childbearing (Moffitt et al., 2011). Fourth, molecular‐genetic studies employing linkage disequilibrium (LD) Score regression (Bulik‐Sullivan et al., 2015) – which uses summary statistics from GWAS to distinguish true genetic associations from confounding factors – have documented genetic correlations between age‐at‐first‐birth and substance involvement (Barban et al., 2016; Liu et al., 2019; Walters et al., 2018). Fifth, evolutionary theorists posit that some forms of antisocial behavior represent variation in life‐history strategy, which implies that genetic variants associated with antisocial behavior should also be associated with accelerated reproduction (Boutwell et al., 2015).

Following from these findings, we tested the hypothesis that a polygenic score for age‐at‐first‐birth would predict disinhibition. Polygenic scores are derived from GWAS and aggregate millions of variants across the genome into a score that indexes an individual's position on a continuum of genetic liability to a trait (Dudbridge, 2013). Our score was derived from the most recent GWAS of age‐at‐first‐birth (Barban et al., 2016). We tested the hypothesis that polygenic influences on age‐at‐first‐birth predict disinhibition in two longitudinal birth cohorts from two countries totaling nearly 3,000 participants. We linked genetic data to self‐reports, informant reports, and official records of disinhibitory problems. Our analysis extended prior research in three ways. First, we tested whether genetic overlap identified in LD Score regression was also identifiable in polygenic prediction of phenotypic outcomes. Second, we included a suite of disinhibitory measures assessed using different methods to test the robustness of associations. Third, our approach studied how associations between the age‐at‐first‐birth polygenic score and disinhibitory problems emerged from childhood into midlife.

**Figure 1:**
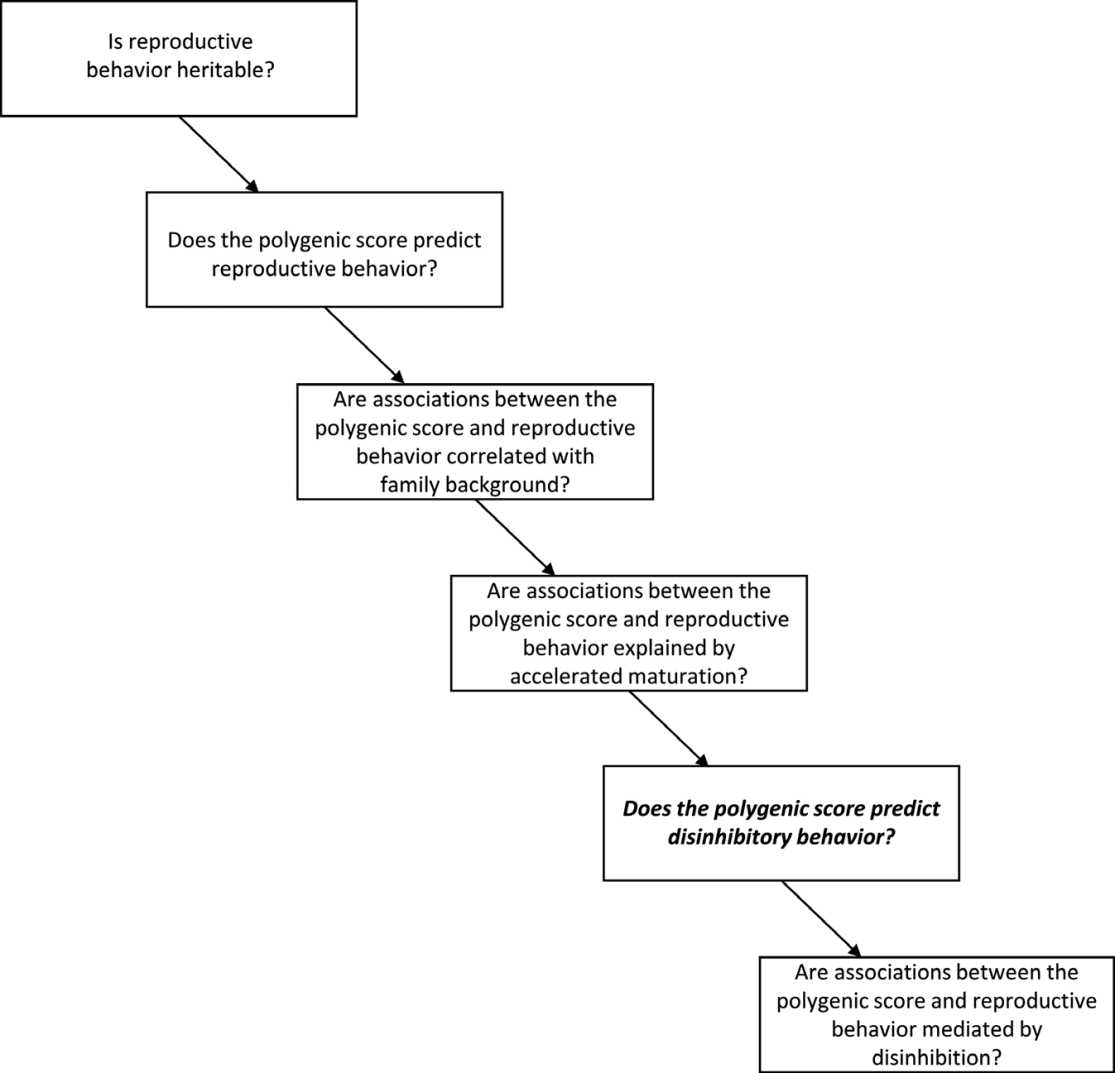
Testing hypotheses about the meaning of a polygenic score for age‐at‐first‐birth. Figure depicts the conceptual framework that guided our sequence of analyses

We conducted a sequence of analyses to set up our test of our primary hypothesis (Figure 1). First, to establish a basis for using the molecular‐genetic polygenic score, we verified that there were quantitative‐genetic influences on reproductive behavior. Second, we tested whether the polygenic score predicted reproductive behaviors including intercourse, pregnancy, and childbearing in our cohorts. Third, we tested whether effects of the score on reproductive behavior were correlated with two familial risk factors for early parenthood: childhood socioeconomic deprivation and early maternal age‐at‐first‐birth. Fourth, we considered another mechanism for effects of the polygenic score on reproductive timing: that it reflects genetic influences on accelerated pubertal maturation. This hypothesis stems from observations that pubertal timing is heritable (Mendle, Turkheimer, & Emery, 2007), youth who reach puberty at younger ages than peers are at greater risk for early pregnancy (Baams, Dubas, Overbeek, & van Aken, 2015), and age‐at‐first‐birth and age‐at‐menarche share common genetic underpinnings (Barban et al., 2016). We tested this hypothesis by including female participants' age‐at‐menarche as an indicator of pubertal timing. Finally, we tested the primary hypothesis that the polygenic score would predict disinhibition. In follow‐up analyses, we tested whether associations between the score and reproductive behavior were mediated by disinhibition.

## Methods

A more detailed description of the study design, measurement, and statistical analysis is included in Appendix S1.

### Samples

#### E‐Risk cohort

Participants in the first cohort were members of the Environmental Risk (E‐Risk) Longitudinal Twin Study, which tracks the development of 2,232 British children born in 1994–1995 in England and Wales (Moffitt & E‐Risk Study Team, 2002). The Joint South London and Maudsley and the Institute of Psychiatry Research Ethics Committee approved each study phase. Parents gave informed consent, and twins gave assent between ages 5 and 12 and informed consent at age 18.

#### Dunedin cohort

Participants in the second cohort were members of the Dunedin Multidisciplinary Health and Development Study, which tracks the development of 1,037 individuals born in 1972–1973 in Dunedin, New Zealand (Poulton, Moffitt, & Silva, 2015). The Otago Ethics Committee approved each phase of the study, and informed consent was obtained from all study members.

### Genotyping and imputation

We used Illumina Omni Express 24 BeadChip arrays (version 1.1; Illumina, Hayward, CA) in the E‐Risk cohort and Illumina HumanOmni Express 12 BeadChip arrays (version 1.1; Illumina, Hayward, CA) in the Dunedin cohort to assay common single‐nucleotide polymorphism (SNP) variation in the genomes of cohort members. We imputed additional SNPs using the IMPUTE2 software (version 2.3.1; Howie, Donnelly, & Marchini, 2009) and the 1,000 Genomes Phase‐3 reference panel (Abecasis et al., 2012). Imputation was conducted on autosomal SNPs appearing in dbSNP (version 140; Sherry et al., 2001) that were ‘called’ in more than 98% of the samples. Invariant SNPs and SNPs with low minor allele frequency (<1%) were excluded. Prephasing and imputation were conducted using a 50‐million‐base‐pair sliding window. The resulting genotype databases included genotyped SNPs and SNPs imputed with 90% probability of a specific genotype among the European‐descent members (90%) of the E‐Risk cohort (*N* = 1,999 participants in 1,011 families) and the non‐Maori members (93%) of the Dunedin cohort (*N* = 918). We analyzed SNPs in Hardy–Weinberg equilibrium (*p* > .01).

### Polygenic scoring

Polygenic scoring was conducted following the method described by Dudbridge (2013) using PRSice (Euesden, Lewis, & O'Reilly, 2015). SNPs reported in the most recent GWAS results released by the Social Science Genetic Association Consortium (Barban et al., 2016) were matched with SNPs in the E‐Risk and Dunedin databases. For each SNP, the count of age‐at‐first‐birth‐associated alleles was weighted according to the effect estimated in the GWAS. Weighted counts were summed across SNPs to compute polygenic scores. We used all matched SNPs to compute polygenic scores irrespective of nominal significance for their association with age‐at‐first‐birth. SNPs were not clumped or pruned for LD prior to analysis. (Polygenic‐score associations computed using different *p‐*value thresholds and clumping are reported in Appendix S2.) Because the majority of associated variants in the GWAS overlapped across men and women, we used the polygenic score computed from the effects for the pooled male–female sample. The cross‐sex and sex‐specific scores showed a similar pattern of associations (Table S1).

Analyses were limited to European‐descent members of the E‐Risk cohort and non‐Maori members of the Dunedin cohort. To control for possible residual population stratification, we conducted a principal components analysis of our genome‐wide SNP database using PLINK (version 1.9; Chang et al., 2015). Analyses were conducted separately in the E‐Risk and Dunedin databases. In the E‐Risk database, one twin was selected at random from each family for principal components analysis. SNP loadings for principal components were applied to co‐twin genetic data to compute principal component values for the full sample. Within each database, we residualized polygenic scores for the first 10 principal components estimated from the genome‐wide SNP data. The residualized score was normally distributed. We standardized residuals (*M* = 0, *SD* = 1) for analysis. We reverse‐coded the score; higher numbers indicate a lower polygenic score (greater genetic risk for early age‐at‐first‐birth).

### Measures

#### Reproductive behaviors

Sexual intercourse, pregnancy, and childbirth were assessed at age 18 in the E‐Risk cohort and through age 38 in the Dunedin cohort. Reproductive behaviors were coded as binary variables to reflect whether participants had met each reproductive milestone by age 18 or younger.

#### Disinhibitory behaviors (Figure 2; Appendix S1)

We measured childhood self‐control between ages 5–10 in the E‐Risk cohort and ages 3–11 in the Dunedin cohort, using a multi‐occasion/multi‐informant strategy. The variable was standardized (*M* = 0, *SD* = 1).

**Figure 2:**
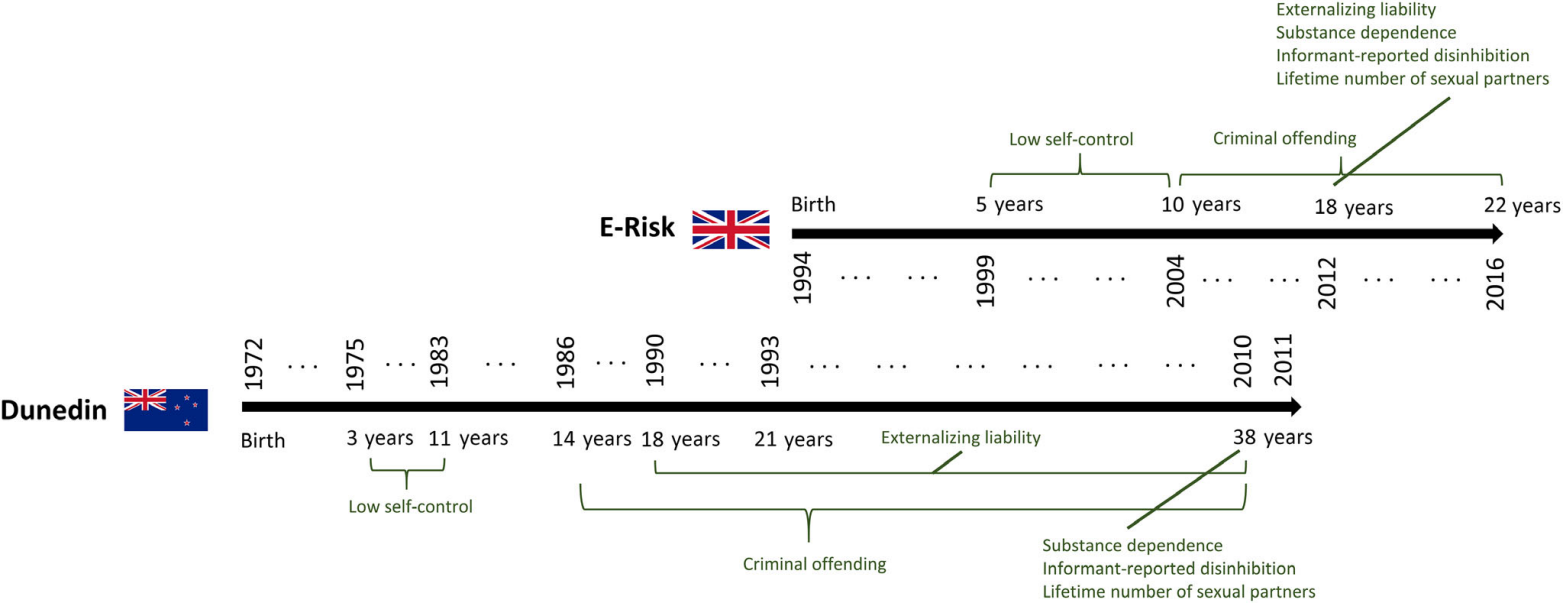
Observation periods for disinhibitory behaviors analyzed in the E‐Risk and Dunedin cohorts

Confirmatory factor analysis was used to derive a factor score (*M* = 0, *SD* = 1) indicating participants' general risk for externalizing psychopathology at age 18 in the E‐Risk cohort and between ages 18–38 in the Dunedin cohort.

Official records of participants' criminal offending were obtained between ages 10–22 in the E‐Risk cohort and ages 14–38 in the Dunedin cohort. Criminal offending was coded as a binary variable to reflect whether participants had a record of an offense.

We measured past‐year DSM‐IV and FTND‐based diagnoses of substance dependence at age 18 in the E‐Risk cohort and age 38 in the Dunedin cohort.

We collected informant reports of participants' disinhibitory problems at age 18 in the E‐Risk cohort and age 38 in the Dunedin cohort. Scores were standardized across informants (*M* = 0, *SD* = 1).

In secondary analyses suggested through peer review, we measured number of lifetime sexual partners at age 18 in the E‐Risk cohort and age 38 in the Dunedin cohort.

#### Family‐context correlates of polygenic risk

In both cohorts, we measured two features of participants' family contexts that might be correlated with effects of the polygenic score on reproductive outcomes: childhood socioeconomic deprivation (standardized to *M* = 0, *SD* = 1) and early maternal age‐at‐first‐birth.

#### Age‐at‐menarche

We tested whether effects of the polygenic score on reproductive behavior were attributable to genetic influences on pubertal timing, measured as female participants' age‐at‐menarche. We did not have a comparable variable for males.

### Statistical analyses

We used a univariate liability threshold model to estimate genetic, shared environmental, and nonshared environmental influences on reproductive behavior in the E‐Risk cohort.

We used Poisson regression models with relative risks (RRs) to test whether the polygenic score predicted reproductive behavior and assess whether childhood socioeconomic deprivation and early maternal age‐at‐first‐birth were correlated with these effects.

We used linear regression to test whether the polygenic score operates through genetic influences on pubertal timing. We tested whether the score predicted female participants' age‐at‐menarche.

We used regression to test our hypothesis that the polygenic score comprises genetic influences on disinhibitory behavior. We analyzed continuously‐distributed outcomes using ordinary least squares. We analyzed binary outcomes using Poisson regression models with RRs. We analyzed count outcomes using negative binomial regression models with incidence rate ratios (IRRs). We used mediation analysis to test whether polygenic‐score associations with reproductive behavior were mediated by disinhibition.

In the E‐Risk cohort, we corrected regression and mediation analyses for the nonindependence of twin observations by clustering standard errors at the family level. In the mediation analyses, 95% confidence intervals were obtained from 500 bootstrap replications. Analyses in which men and women were combined were adjusted for sex. Analyses in which E‐Risk and Dunedin participants were combined were adjusted for cohort/study. Analyses were conducted using SAS v9.4 (SAS Institute Inc., Cary, NC) and Mplus v7 (Muthén & Muthén, 1998–2015).

## Results

### Are genetic influences on reproductive behavior apparent in the E‐Risk cohort?

We first used the E‐Risk cohort to replicate prior findings of quantitative‐genetic influences on reproductive behavior, to establish that there was a basis to proceed with testing for an association between the molecular‐genetic polygenic score and reproductive outcomes. A liability threshold model indicated that 64% of the variance in adolescent sexual intercourse was attributable to genetic factors (95% CI = 34–93), 20% was attributable to shared environmental factors (95% CI = 0–48), and 16% was attributable to unique environmental factors and measurement error (95% CI = 10–23). (Shared environmental influences could be constrained to zero without a significant decrement in model fit (χ^2^ = 2.03, *df* = 1, *p* = .154.) These estimates are within the range of those reported in prior studies (see Harden, 2014). Due to the small number of E‐Risk participants who reported having a pregnancy or child at age 18 or younger, we did not fit biometric models for these outcomes. However, previous research has established that pregnancy and childbearing are under genetic influence (Harden, 2014).

### Do young people's polygenic scores predict their reproductive behaviors?

We next tested whether the polygenic score predicted three reproductive behaviors: sexual intercourse, pregnancy, and childbirth. Given the low prevalences of early pregnancy and childbearing, we pooled participants across cohorts. Participants with lower polygenic scores were more likely to have intercourse and become pregnant by age 18: A standard‐deviation decrease in the score was associated with a 6% increase in the likelihood of having intercourse (RR = 1.06, 95% CI = 1.04–1.09) and a 25% increase in the likelihood of becoming pregnant (RR = 1.25, 95% CI = 1.12–1.41; Table 1). The association with participants' age‐at‐first‐birth was not statistically significant, likely due to the relatively small number of individuals who became parents by age 18 (*n* = 76; RR = 1.25, 95% CI = 0.99–1.59; Table 1). However, the association was in the expected direction and consistent in magnitude with what is reported in the literature (Table S2; Figure S1).

**Table 1:** Risk ratios for associations between a polygenic score for age‐at‐first‐birth and reproductive behaviors

Outcome^a^	Pooled E‐Risk and Dunedin cohorts (N* = *2,917)											
	Analytic *N*	Cases (%)	Baseline	*p‐*Value^b^	Cohen's *d* ^c^	Pearson's *r* ^c^	Adjusted for SES	*p‐*Value	Adjusted for maternal AFB	*p‐*Value	Adjusted for SES and maternal AFB	*p‐*Value
Intercourse	2,736	1,982 (72.4)	1.06 [1.04–1.09]	<.001	.21	.10	1.05 [1.03–1.08]	<.001	1.05 [1.02–1.07]	<.001	1.04 [1.02–1.07]	<.001
Pregnancy	2,734	248 (9.1)	1.25 [1.12‐1.41]	<.001	.17	.08	1.17 [1.04–1.32]	.008	1.14 [1.02–1.28]	.024	1.12 [1.00–1.26]	.057
Birth	2,737	76 (2.8)	1.25 [0.99–1.59]	.065	.08	.04	1.12 [0.88–1.42]	.355	1.10 [0.88–1.39]	.402	1.05 [0.83–1.33]	.684

The polygenic score was reverse‐coded. Analyses were restricted to the Caucasian samples (E‐Risk: *N* = 1,999, Dunedin: *N* = 918). Models controlled for sex and cohort/study. Brackets indicate 95% confidence intervals. AFB, age‐at‐first‐birth; SES, childhood socioeconomic status.

^a^ Binary variables, coded to reflect whether participants had met each reproductive milestone by age 18 or younger.

^b^ Our analysis prioritizes the reporting of effect sizes and their uncertainty; however, we also report *p*‐values for full disclosure. The Bonferroni‐adjusted *p*‐value for tests of baseline associations is .017.

^c^ Average effect size across three approaches used to account for the clustering of E‐Risk twins within families. All approaches produced very similar estimates, available on request from the corresponding author.

### Are the effects of individuals' polygenic scores on their reproductive behaviors correlated with their family backgrounds?

In the pooled E‐Risk and Dunedin cohorts, we next tested whether the effects of participants' polygenic scores on their reproductive behaviors were correlated with two features of their family backgrounds: socioeconomic deprivation and a family history of early childbearing (early maternal age‐at‐first‐birth). We selected these measures because they are established risk factors for early reproductive behavior.

Participants who grew up in more socioeconomically deprived homes and had mothers with earlier ages‐at‐first‐birth were at increased risk for early reproductive behavior (Table 2). We also observed an association between participants' polygenic scores and these features of their family backgrounds: Participants with lower polygenic scores were more likely to have grown up in socioeconomically deprived households (β = .18, 95% CI = .14–.22) and have mothers who gave birth to their first child at an early age (β = .15, 95% CI = .10–.19). After adjusting for these measures of family context, associations between the polygenic score and reproductive behaviors were attenuated (intercourse: RR = 1.04, 95% CI = 1.02–1.07; pregnancy: RR = 1.12, 95% CI = 1.00–1.26; childbirth: RR = 1.05, 95% CI = 0.83–1.33; Table 1).

**Table 2:** Risk ratios for associations between measures of family background and reproductive behaviors

Outcome^a^	Pooled E‐Risk and Dunedin cohorts (*N = *2,917)				
	*N* ^b^	Socioeconomic deprivation	*p‐*Value	Early maternal age‐at‐first‐birth	*p‐*Value
Intercourse	2,732|2,724	1.07 [1.04–1.10]	<.001	1.03 [1.02–1.03]	<.001
Pregnancy	2,730|2,721	1.61 [1.42–1.84]	<.001	1.15 [1.11–1.19]	<.001
Birth	2,733|2,724	2.31 [1.73–3.10]	<.001	1.19 [1.11–1.28]	<.001

The polygenic score was associated with childhood socioeconomic deprivation (β = .18, 95% CI = .14–.22, *p* < .001) and maternal age‐at‐first‐birth (β = .15, 95% CI = .10–.19, *p* < .001). Models controlled for sex and cohort/study. Brackets indicate 95% confidence intervals.

^a^ Binary variables, coded to reflect whether participants had met each reproductive milestone by age 18 or younger.

^b^ Ns indicate the number of participants with data for both the reproductive outcome and childhood socioeconomic deprivation (before the line) and maternal age‐at‐first‐birth (after the line).

Sibling‐difference analysis is another way to adjust for familial influences on associations between the polygenic score and reproductive behavior. Sibling‐difference analysis controls for influences on reproductive behavior shared by siblings reared in the same household. We conducted a sibling‐difference analysis in the E‐Risk cohort, using mixed‐effects models that parsed polygenic‐score effects into within‐sibling‐pair and between‐sibling‐pair effects. Because MZ twins are genetically identical, the analysis was limited to DZ twins (correlation of DZ twins' polygenic scores: *r = *.54, *p < *.001). Consistent with the attenuated associations following covariate adjustment, polygenic‐score associations with reproductive behavior within DZ pairs were also attenuated (Table S3). These findings indicate a gene–environment correlation: Children inherit genes that increase risk for early childbearing, and these genes are correlated with social environments that also increase risk for early childbearing.

### Are the effects of individuals' polygenic scores on their reproductive behaviors explained by accelerated puberty?

Individuals with lower polygenic scores for age‐at‐first‐birth may exhibit earlier reproductive behaviors in part because they are at greater genetic risk for accelerated maturation (Baams et al., 2015). We tested this possibility in the pooled E‐Risk and Dunedin cohorts, using female participants' age‐at‐menarche as an indicator of pubertal timing. Women with lower polygenic scores were not more likely to start menstruating at an early age. This was the case both in a linear regression model (β = −.05, 95% CI = −.10–.01) and in a proportional hazards regression model (hazard ratio = 1.03, 95% CI = 0.98–1.09).^1001^ Further, associations between the polygenic score and reproductive behaviors were unchanged when age‐at‐menarche was included in the model (Table S4). We therefore turned our attention to the hypothesis that the polygenic‐score associations with reproductive behavior reflect genetic influences on disinhibitory behavior.

### Do individuals' polygenic scores predict their disinhibitory behaviors through adolescence?

In the E‐Risk cohort, we tested our hypothesis that the age‐at‐first‐birth polygenic score predicts disinhibition. Results supported this hypothesis: Primary analyses indicated that participants with lower scores were at increased risk for a series of disinhibitory behaviors across their early lives. In childhood, they had poorer self‐control (β = .07, 95% CI = .02–.12). In adolescence, they were at greater risk for externalizing psychopathology (β = .08, 95% CI = .03–.13), were more likely to have a criminal record (RR = 1.16, 95% CI = 1.01–1.33), and had higher rates of substance dependence (RR = 1.14, 95% CI = 1.04–1.24). These associations were verified by people whom study members had nominated as informants who knew them well. Individuals with lower polygenic scores were rated by informants as having more disinhibitory problems (β = .07, 95% CI = .02–.12; Table 3).^1002^ Secondary analyses indicated that participants with lower polygenic scores had more lifetime sexual partners (IRR = 1.12, 95% CI = 1.06–1.18).

**Table 3:** Associations between polygenic scores for age‐at‐first‐birth and disinhibitory behaviors in two birth cohorts

Outcome	Effect size	E‐Risk cohort (*N* = 1,999)							Dunedin cohort (*N* = 918)						
		Age assessed (years)	*N*	Mean [*SD*] or *n* (%)	Association	*p‐*Value^a^	Cohen's *d* ^b^	Pearson's *r* ^b^	Age assessed (years)	*N*	Mean [*SD*] or *n* (%)	Association	*p‐*Value^a^	Cohen's *d*	Pearson's *r*
Primary outcomes^c^															
Low childhood self‐control^d^	β	5–10	1,999	0.01 [0.99]	.07 [.02–.12]	.006	.15	.08	3–11	918	−0.02 [0.95]	.12 [.06–.18]	<.001	.25	.13
Externalizing liability^e^	β	18	1,863	0.01 [1.00]	.08 [.03–.13]	.004	.17	.08	18–38	918	−0.03 [0.98]	.08 [.02–.14]	.015	.16	.08
Criminal offending	RR	10–22	1,857	*n* = 208 (11.2%)	1.16 [1.01–1.33]	.039	.12	.06	14–38	898	*n* = 238 (26.5%)	1.18 [1.04–1.34]	.010	.17	.09
Substance dependence	RR	18	1,860	*n* = 413 (22.2%)	1.14 [1.04–1.24]	.006	.16	.08	38	886	*n* = 165 (18.6%)	1.19 [1.02–1.38]	.028	.15	.07
Informant‐reported disinhibition^f^	β	18	1,851	0.01 [1.01]	.07 [.02–.12]	.007	.16	.08	38	873	−0.02 [0.98]	.02 [−.04–.09]	.501	.05	.02
Secondary outcome^c^															
Lifetime number of sexual partners	IRR	18	1,810	1.90 [2.00]	1.12 [1.06–1.18]	<.001	.25	.12	38	914	17.39 [15.18]	1.07 [1.01–1.14]	.016	.16	.08

The polygenic score was reverse‐coded. Analyses were restricted to the Caucasian samples (E‐Risk: *N* = 1,999, Dunedin: *N* = 918). Models controlled for sex. Brackets indicate 95% confidence intervals. β, standardized regression coefficient; IRR, incidence rate ratio; RR, risk ratio.

^a^ Our analysis prioritizes the reporting of effect sizes and their uncertainty; however, we also report *p*‐values for full disclosure. Within each cohort, the Bonferroni‐adjusted *p*‐value for tests of associations with primary outcomes is .010.

^b^ Average effect size across three approaches used to account for the clustering of twins within families. All approaches produced very similar estimates, available on request from the corresponding author.

^c^ Primary outcomes were prespecified. Secondary outcome was added in response to peer review.

^d^ The self‐control factor was standardized (*M* = 0, *SD* = 1) within the full samples (E‐Risk: *N* = 2,232, Dunedin: *N* = 1,037). Higher scores indicate lower levels of self‐control.

^e^ The externalizing liability factor was standardized (*M* = 0, *SD* = 1) within the full sample of E‐Risk participants at age 18 (*N* = 2,066) and the full sample of Dunedin participants with externalizing data for at least one assessment between ages 18 and 38 (*N* = 1,000).

^f^ Informant‐reported disinhibition was standardized (*M* = 0, *SD* = 1) within the full samples of participants with informant‐report data (E‐Risk: *N* = 2,052, Dunedin: *N* = 934).

### Do individuals' polygenic scores predict their disinhibitory behaviors into midlife?

We replicated our test of the hypothesis in the Dunedin cohort, using the same disinhibitory outcomes measured into middle adulthood. Consistent with findings in the E‐Risk cohort, primary analyses showed that Dunedin participants with lower polygenic scores were at increased risk for a series of disinhibitory behaviors. They had poorer childhood self‐control (β = .12, 95% CI = .06–.18), were at elevated risk for externalizing psychopathology between ages 18 and 38 (β = .08, 95% CI = .02–.14), were more likely to have been convicted of a crime by age 38 (RR = 1.18, 95% CI = 1.04–1.34), and had higher rates of substance dependence at age 38 (RR = 1.19, 95% CI = 1.02–1.38). The only exception to our replication of primary analyses was that Dunedin participants' polygenic scores were not associated with informants' ratings of their disinhibitory problems (β = .02, 95% CI = −.04–.09; Table 3).^2^ Consistent with findings in the E‐Risk cohort, secondary analyses showed that participants with lower polygenic scores had more lifetime sexual partners (IRR = 1.07, 95% CI = 1.01–1.14).

### Are the effects of individuals' polygenic scores on their reproductive behaviors mediated by disinhibition?

In both cohorts, there was support for our hypothesis that the age‐at‐first‐birth polygenic score captures disinhibition. In the pooled E‐Risk and Dunedin cohorts, we further tested whether disinhibition mediated the effects of the score on reproductive behaviors, using our measure of childhood self‐control. As noted above, participants with lower polygenic scores had lower self‐control. Further, participants with lower self‐control were more likely to have intercourse (RR = 1.08, 95% CI = 1.05–1.10), become pregnant (RR = 1.48, 95% CI = 1.33–1.64), and become parents at an early age (RR = 1.51, 95% CI = 1.27–1.79). Low childhood self‐control was a statistically significant mediator of all genetic associations with reproductive behaviors. It explained 11%, 16%, and 25% of the associations with intercourse, pregnancy, and childbirth, respectively (Table S5). The estimate for childbirth should be interpreted with caution, however, as the total effect for the model was not statistically significant.

## Discussion

In two prospective birth cohorts from two countries, we tested the hypothesis that a polygenic score for age‐at‐first‐birth would predict disinhibition. In both cohorts, participants with lower polygenic scores had poorer childhood self‐control, were at elevated risk for externalizing psychopathology, were more likely to have a criminal record, had higher rates of substance dependence, and had more lifetime sexual partners. (Polygenic‐score associations with informant reports of disinhibitory problems did not replicate across cohorts, possibly due to differences in assessment age or informant type.) Childhood disinhibitory problems preceded the onset of sexual activity, which helped rule out the possibility of reverse causation (that early pregnancy led to disinhibitory behavior). Further, childhood disinhibition partly mediated associations between the polygenic score and reproductive outcomes. In contrast, the score was not linked to accelerated puberty. These results suggest that genetic discoveries for age‐at‐first‐birth are about more than reproductive biology. They are also about the psychological traits and behaviors that accompany early parenthood.

Four design features bolster the substance of these findings. First, we replicated analyses across two population‐representative cohorts that collectively spanned developmental periods from childhood to midlife. Second, we replicated analyses in twins and singletons, helping to allay concerns about generalizability of findings from twins. Third, both cohorts have high retention rates (E‐Risk = 93%, Dunedin = 95%), reducing potential for bias when analyzing behaviors that predict attrition, such as offending. Fourth, associations were present across different disinhibitory behaviors assessed using different methods.

We acknowledge limitations. First, findings cannot be generalized to individuals of non‐European ancestry. Second, follow‐up of disinhibitory behaviors was right‐censored at midlife. Third, associations may have differed before the availability of birth control. Fourth, our analysis centered on disinhibitory outcomes, and the polygenic score might predict psychopathology more broadly. However, the score did not predict neuroticism (Appendix S3). Fifth, findings concerning pubertal maturation are limited to females, as we did not have a comparable measure in males. Sixth, although we did not observe a statistically‐significant association between the polygenic score and age‐at‐menarche, other studies have (Gaydosh, Belsky, Domingue, Boardman, & Harris, 2018), and studies have shown genetic correlations between age‐at‐first‐birth and age‐at‐menarche (Barban et al., 2016; Liu et al., 2019). Finally, effect sizes for polygenic‐score associations were very modest (Tables 1 and 3).

With limitations in mind, several implications can be noted. First, our findings align with prior reports showing that genetic discoveries for social and behavioral phenotypes (e.g., educational attainment) predict outcomes beyond the target phenotype (Belsky et al., 2016; Wertz et al., 2018). This highlights the ubiquity of pleiotropy and indicates an opportunity to delve deeper into the meaning of pleiotropic effects. What biological, psychological, and behavioral characteristics connect genomic discoveries with different outcomes? Future research should aim to clarify the mechanisms underlying pleiotropic associations. Our results also point to the utility of proxy phenotyping. Previous studies have shown that easy‐to‐measure phenotypes (e.g., education) can be used to help identify the genetic architecture of traits that are more difficult to assess (e.g., cognitive ability; Rietveld et al., 2014). Our study suggests this principle may apply more broadly. Age‐at‐first‐birth may be a useful proxy phenotype for researchers interested in disinhibition. Multivariate genomic methods such as Genomic Structural Equation Modeling (Grotzinger et al., 2019) offer the opportunity to explore the genetic variation that connects and differentiates these traits. Such work can help characterize the nomological net in which the genetics of reproductive behavior are embedded. It can also foster interdisciplinary collaboration: Interest in reproductive and disinhibitory behavior unites the social and behavioral sciences.

Second, polygenic‐score associations with reproductive behaviors were attenuated after accounting for features of participants' family backgrounds, including their social‐class origins. This joins a growing body of findings (e.g., Belsky et al., 2019; Krapohl & Plomin, 2016) showing that genetics discovered in GWAS of social science outcomes are correlated with socioeconomic and other environmental measures. Such gene–environment correlations are important for two reasons. First, they indicate that there exist challenges to establishing causality with genetic data, just as there exist challenges to establishing causality with phenotypic data. Second, they indicate opportunities for researchers who incorporate environmental measures into genetic designs. These include improving inferences about the magnitude of direct genetic effects, identifying mechanisms of gene–environment interplay, and testing how genetic effects may operate through environments [e.g., via social‐genetic processes (Kong et al., 2018; Wertz et al., 2019)].

Third, beyond cranking the sample‐size handle, genetics researchers should attend to *who* is represented in genetic discovery samples. For researchers interested in using polygenic scores to conduct cross‐phenotype prediction, this question is particularly relevant. For instance, GWAS of age‐at‐first‐birth may experience selective nonparticipation by individuals who exhibit the most disinhibitory problems: teen parents. Indeed, the mean age‐at‐first‐birth among participants in the GWAS from which we derived our polygenic score (Barban et al., 2016) was 26.8 years, which is beyond the period of peak prevalence for disinhibitory behavior. It is notable that despite this under‐representation of young parents – which should bias the GWAS signal away from disinhibition – we still observed a stronger association between the polygenic score and disinhibition relative to pubertal timing. Much emphasis has been placed on the importance of increasing GWAS sample sizes. The predictive power of polygenic scores for proxy phenotypes such as age‐at‐first‐birth may also be increased by maximizing representation of populations who best capture the phenotype of interest.

Finally, these results build on prior literature (e.g., Hoffman & Maynard, 2008) showing that young parents and their children carry a unique set of vulnerabilities. Our findings do not suggest that genes determine reproductive and disinhibitory outcomes. These behaviors are also influenced by the social environment. Further, age‐at‐first‐birth polygenic scores are currently far from sensitive or specific enough to forecast behaviors with any reasonable degree of precision. However, efforts to support young parents can be strengthened by taking into account the social *and* genetic context in which early childbearing occurs. The demands of child‐rearing may pose a particular challenge to individuals at elevated risk for emotional and behavioral dysregulation. Alongside established interventions for young parents (e.g., nurse home‐visiting programs; Hodgkinson, Beers, Southammakosane, & Lewin, 2014), interventions to improve self‐control and reduce antisocial behavior and substance misuse (Pandey et al., 2018; Piquero, Jennings, Farrington, Diamond, & Gonzalez, 2016; Stockings et al., 2016) may reduce liability to mental‐health difficulties. Further, genetic risk for disinhibitory problems manifests early in life: We observed polygenic‐score associations with self‐control in childhood. Improving children's self‐regulation abilities and monitoring vulnerable youth for disinhibitory problems might mitigate risk for early childbearing.

## Conclusion

This study aimed to clarify the meaning of new molecular‐genetic discoveries for reproductive behavior. A polygenic score for age‐at‐first‐birth predicts an array of disinhibitory problems, which are evident in childhood and persist into midlife. This novel finding extends prior research in the health and behavioral sciences, by showing how discoveries about the genetics of reproductive behavior (a) provide insights about constructs (such as self‐regulation) that are of interest to child psychologists and psychiatrists, (b) shed light on intra‐ and intergenerational developmental processes, and (c) need to be interpreted with caution in relation to the contexts in which they are observed.

## Supporting information


**Appendix S1.** Supplementary methods.
**Appendix S2** **.** Associations between the age‐at‐first‐birth polygenic score and two disinhibitory outcomes in the Dunedin cohort, using (a) clumping to account for linkage disequilibrium and (b) different *p*‐value thresholds for SNP inclusion.
**Appendix S3** **.** Associations between the age‐at‐first‐birth polygenic score and informant‐reported Neuroticism.
**Table S1** **.** Comparison of cross‐sex and sex‐specific polygenic scores for age‐at‐first‐birth.
**Table S2** **.** Results from survival models testing polygenic‐score associations with continuously‐coded reproductive outcomes.
**Table S3** **.** DZ twin‐difference analysis of polygenic‐score associations in the E‐Risk cohort.
**Table S4** **.** Associations between the age‐at‐first‐birth polygenic score and reproductive and disinhibitory behaviors among women, controlling for age‐at‐menarche.
**Table S5** **.** Does childhood disinhibition mediate associations between the polygenic score and reproductive behaviors?
**Figure S1** **.** Polygenic prediction effect sizes for the age‐at‐first‐birth score across different cohorts.

## Notes

^1.^ We also tested associations between the polygenic score and age‐at‐menarche within each cohort. Associations were not statistically significant (E‐Risk: β = −.06, 95% CI = −.13–.01; Dunedin: β = −.02, 95% CI = −.11–.08).

^2.^ Among women, associations between the polygenic score and disinhibitory behaviors were unchanged when age‐at‐menarche was included in the model, providing further evidence that the score did not operate through accelerated puberty (Table S4).
