# Supplementary material for: A polygenic score for age‐at‐first‐birth predicts disinhibition
Source: J Child Psychol Psychiatry. 2020 Mar 27;61(12):1349–59. doi: 10.1111/jcpp.13224 (PMC7529719; doi:10.1111/jcpp.13224)
Supplement: Supplementary file 1 — Appendix S1. Supplementary methods. Appendix S2 . Associations between the age‐at‐first‐birth polygenic score and two disinhibitory outcomes in the Dunedin cohort, using (a) clumping to account for linkage disequilibrium and (b) different p‐value thresholds for SNP inclusion. Appendix S3 . Associations between the age‐at‐first‐birth polygenic score and informant‐reported Neuroticism. Table S1 . Comparison of cross‐sex and sex‐specific polygenic scores for age‐at‐first‐birth. Table S2 . Results from survival models testing polygenic‐score associations with continuously‐coded reproductive outcomes. Table S3 . DZ twin‐difference analysis of polygenic‐score associations in the E‐Risk cohort. Table S4 . Associations between the age‐at‐first‐birth polygenic score and reproductive and disinhibitory behaviors among women, controlling for age‐at‐menarche. Table S5 . Does childhood disinhibition mediate associations between the polygenic score and reproductive behaviors? Figure S1 . Polygenic prediction effect sizes for the age‐at‐first‐birth score across different cohorts. [file JCPP-61-1349-s001.docx]

**A polygenic score for age-at-first-birth predicts disinhibition**

**Leah S. Richmond-Rakerd et al.**

**Supplementary Information**

**Appendix S1.** ..... 2

**Appendix S2.** …. 9

**Appendix S3.** …. 12

**Table S1.** ……. 13

**Table S2.** ……. 14

**Table S3.** ……. 15

**Table S4.** ……. 16

**Table S5.** ……. 17

**Figure S1.** ……. 18

**Supplementary References.**  19

**Appendix S1.** Supplementary methods.

**Samples**

**Environmental Risk (E-Risk) cohort.** Participants in the first cohort were members of the Environmental Risk (E-Risk) Longitudinal Twin Study, a birth cohort of 2,232 British children drawn from a larger register of twins born in England and Wales in 1994-1995 (Trouton, Spinath, & Plomin, 2002). Details are reported elsewhere (Moffitt & E-Risk Study Team, 2002). The E-Risk sample was constructed in 1999-2000, when 1,116 families (93% of those eligible) with same-sex 5-year-old twins participated in home-visit assessments. This sample comprised 56% monozygotic (MZ) and 44% dizygotic (DZ) twin pairs; sex was evenly distributed within zygosity (49% male). Families were recruited to represent the UK population with newborns in the 1990s on the basis of residential location throughout England and Wales and mother’s age. Teenaged mothers with twins were over-selected to replace high-risk families selectively lost to the register through non-response. Older mothers having twins via assisted reproduction were under-selected to avoid an excess of well-educated older mothers. The study sample represented the full range of socioeconomic conditions in the UK, as reflected in families’ distribution on a neighborhood-level socioeconomic index (Odgers, Caspi, Bates, Sampson, & Moffitt, 2012): 25.6% of E-Risk families live in “wealthy achiever” neighborhoods compared to 25.3% nationwide; 5.3% vs. 11.6% live in “urban prosperity” neighborhoods; 29.6% vs. 26.9% live in “comfortably off” neighborhoods; 13.4% vs. 13.9% live in “moderate means” neighborhoods, and 26.1% vs. 20.7% live in “hard-pressed” neighborhoods. E-Risk underrepresents “urban prosperity” households because they are likely to be childless.

Follow-up home visits took place when study participants were aged 7 (98% participation), 10 (96%), 12 (96%), and 18 years (93% participation). Home visits at ages 5-12 assessed twin participants and their mothers; only twins were assessed at age 18.

Each twin was assessed by a different interviewer. Data are supplemented by searches of official records and questionnaires that are mailed, as developmentally appropriate, to teachers, and co-informants nominated by participants. The Joint South London and Maudsley and the Institute of Psychiatry Research Ethics Committee approved each study phase. Parents gave informed consent and twins gave assent between 5-12 years and then informed consent at age 18.

**Dunedin cohort.** Participants in the second cohort were members of the Dunedin Multidisciplinary Health and Development Study, a longitudinal investigation of health and behavior in a complete birth cohort. Dunedin participants (N=1,037, 91% of eligible births, 52% male) were all individuals born between April 1972 and March 1973 in Dunedin, New Zealand, who were eligible based on residence in the province at age 3 years and who participated in the first assessment at age 3 years. Details are reported elsewhere (Poulton, Moffitt, & Silva, 2015). The cohort represented the full range of socioeconomic status in the general population of New Zealand's South Island. On adult health, the cohort matches the New Zealand National Health and Nutrition Survey on key health indicators (e.g. body mass index, smoking, visits to the doctor) and matches the New Zealand Census of people the same age on educational attainment (Richmond-Rakerd et al., in press).

Assessments were performed at birth and ages 3, 5, 7, 9, 11, 13, 15, 18, 21, 26, 32, and, most recently, 38 years, when 1,007 participants were still alive, with 95% retention. At each assessment wave, participants are brought to the Dunedin research unit for a full day of interviews and examinations. These data are supplemented by searches of official records and by questionnaires that are mailed, as developmentally appropriate, to parents, teachers, and peers nominated by the participants themselves. The Otago Ethics Committee approved each phase of the study and informed consent was obtained from all study members.

**Genotyping and imputation**

We used Illumina Omni Express 24 BeadChip arrays (Version 1.1; Illumina, Hayward, CA) in the E-Risk cohort and Illumina HumanOmni Express 12 BeadChip arrays (Version 1.1; Illumina, Hayward, CA) in the Dunedin cohort to assay common single-nucleotide polymorphism (SNP) variation in the genomes of cohort members. We imputed additional SNPs using the IMPUTE2 software (Version 2.3.1; <https://mathgen.stats.ox.ac.uk/impute/impute_v2.html>; Howie, Donnelly, & Marchini, 2009) and the 1000 Genomes Phase 3 reference panel (Abecasis et al., 2012). Imputation was conducted on autosomal SNPs appearing in dbSNP (Version 140; http://www.ncbi.nlm.nih.gov/SNP/; Sherry et al., 2001) that were “called” in more than 98% of the samples. Invariant SNPs and SNPs with low minor allele frequency (<1%) were excluded. The E-Risk cohort contains monozygotic twins, who are genetically identical; we therefore empirically measured genotypes of one randomly-selected twin per pair and assigned these data to their monozygotic co-twin. MZ status was confirmed using genotypic data and SNPs from DNA methylation data for subsets of the sample. We directly measured genotypes of both members of dizygotic twin pairs. Prephasing and imputation were conducted using a 50-million-base-pair sliding window. The resulting genotype databases included genotyped SNPs and SNPs imputed with 90% probability of a specific genotype among the European-descent members (90%) of the E-Risk cohort (N=1,999 participants in 1,011 families) and the non-Maori members (93%) of the Dunedin cohort (N=918). Ancestry was determined using self-reports. We accounted for potential remaining differences in ancestry through principal components analysis of the genetic data (details below). We analyzed SNPs in Hardy-Weinberg equilibrium (p>.01).

**Polygenic scoring**

Polygenic scoring was conducted following the method described by Dudbridge (Dudbridge, 2013) using PRSice (Euesden, Lewis, & O'Reilly, 2015). Briefly, SNPs reported in the most recent GWAS results released by the Social Science Genetic Association Consortium (Barban et al., 2016) were matched with SNPs in the E-Risk and Dunedin databases. For each SNP, the count of age-at-first birth-associated alleles was weighted according to the effect estimated in the GWAS. Weighted counts were summed across SNPs to compute polygenic scores. We used all matched SNPs to compute polygenic scores irrespective of nominal significance for their association with age-at-first birth. SNPs were not clumped or pruned for LD prior to analysis (Ware et al., 2017). Debate over best practices for polygenic-score calculation is ongoing and papers in the literature use different approaches. Given this, we also report polygenic-score associations computed using different *p-*value thresholds and clumping (**Appendix S2**). Because the majority of associated variants in the GWAS overlapped across men and women, we used the polygenic score computed from the effects for the pooled male-female sample. The cross-sex and sex-specific scores showed a similar pattern of associations (**Table S1**).

Analyses were limited to the European-descent members of the E-Risk cohort and the non-Maori members of the Dunedin cohort. However, polygenic-score values may be influenced by subtle differences in ancestry, even among European-descent individuals. To control for possible residual population stratification, we conducted a principal components analysis of our genome-wide SNP database using PLINK (Version 1.9; Chang et al., 2015). Analyses were conducted separately in the E-Risk and Dunedin databases. In the E-Risk database, one twin was selected at random from each family for principal components analysis. SNP-loadings for principal components were applied to co-twin genetic data to compute principal component values for the full sample.

Within each database, we residualized polygenic scores for the first 10 principal components estimated from the genome-wide SNP data. The residualized score was normally distributed. We standardized residuals (M=0, SD=1) for analysis. We reverse-coded the score, so that higher numbers indicate a lower polygenic score for age-at-first-birth (greater genetic risk for early age-at-first-birth).

**Measures**

The following measures were collected in both cohorts:

| **Measure** | **E-Risk cohort** | **Dunedin cohort** |
| --- | --- | --- |
| ***Reproductive behaviors*** | Reproductive behaviors were assessed at age 18 using a computer interview. They were coded as binary variables to reflect whether participants had met each reproductive milestone by age 18 years or younger. We coded reproductive behaviors as binary variables because due to the relatively young age of assessment, there was insufficient variation in age-at-first-pregnancy or age-at-first-birth to code them on continuous scales. | Reproductive behaviors were assessed through age 38 using a computer interview and a life-history calendar. We used Dunedin participants’ reports about the age at which they met each reproductive milestone to age-match the assessments across cohorts. Consistent with the approach used in the E-Risk cohort, reproductive behaviors were coded as binary variables to reflect whether participants had met each reproductive milestone by age 18 years or younger. |
| **Sexual intercourse** | 1,238/1,834 (67.5%) of participants reported having had sexual intercourse by age 18. | 744/902 (82.5%) of participants reported having had sexual intercourse by age 18. |
| **Pregnancy** | 146/1,825 (8.0%) of participants reported having been pregnant (females) or having made someone pregnant (males) by age 18. | 102/909 (11.2%) of participants reported having been pregnant (females) or having made someone pregnant (males) by age 18. |
| **Childbirth** | 41/1,819 (2.3%) of participants reported having had a child by age 18. | 35/918 (3.8%) of participants reported having had a child by age 18. |
|  |  |  |
| ***Disinhibitory behaviors*** |  |  |
| **Low childhood self-control** | Low childhood self-control was measured using a multi-occasion/multi-informant strategy (Wertz et al., 2018). A self-control factor was estimated via multiple measures, including observational ratings of participants’ lack of control (age 5 years), parent and teacher reports of poor impulse control (ages 5, 7 and 10 years), self-reports of inattentive and impulsive behavior (age 7 years), and interviewer judgments of the personality trait of conscientiousness (age 10 years). Based on principal components analysis, the standardized measures were averaged into a single composite score (M=0, SD=1). | Low childhood self-control was measured using multiple measures of self-control: observational ratings of participants’ lack of control (ages 3 and 5) and parent, teacher, and self-reports of impulsive aggression, overactivity, lack of persistence, inattention, and impulsivity (ages 5, 7, 9, and 11; Moffitt et al., 2011). Based on principal components analysis, the standardized measures were averaged into a single composite score (M=0, SD=1) |
| **Externalizing liability** | Participants’ liability to externalizing psychopathology was measured at age 18. Details are provided in a previous publication (Schaefer et al., 2018). Briefly, we assessed past-year symptoms of five disorders: DSM-IV symptoms of alcohol dependence and cannabis dependence were assessed via the Diagnostic Interview Schedule (DIS; Robins, Cottler, Bucholz, & Compton, 1995); conduct disorder was measured by inquiring about DSM-IV symptoms (American Psychiatric Association, 1994); symptoms of tobacco dependence were assessed with the Fagerström Test for Nicotine Dependence (FTND; Heatherton, Kozlowski, Frecker, & Fagerström, 1991); and attention-deficit/hyperactivity disorder was measured by inquiring about DSM-5 symptoms (Agnew-Blais et al., 2016). Confirmatory factor analysis was used to derive a factor score (M=0, SD=1) indicating individuals’ general risk for externalizing psychopathology in adolescence. | Participants’ liability to externalizing psychopathology was measured at ages 18, 21, 26, 32, and 38. Details are provided in a previous publication (Caspi et al., 2014). Briefly, we repeatedly assessed past-year symptoms of five disorders: DSM symptoms of alcohol dependence, cannabis dependence, hard drug dependence, and conduct disorder were assessed via the DIS; and symptoms of tobacco dependence were assessed with the FTND. Confirmatory factor analysis was used to derive a factor score (M=0, SD=1) indicating individuals’ general risk for externalizing psychopathology from adolescence to midlife. |
| **Criminal offending** | Official records of participants’ criminal offending were obtained through UK Police National Computer record searches conducted in cooperation with the UK Ministry of Justice. Records include complete histories of cautions and convictions beginning at age 10, the age of criminal responsibility. Our data are complete through age 22. Criminal offending was coded as a binary variable to reflect whether participants had been cautioned or convicted. 208/1,857 (11.2%) of participants had an official record of a criminal offense. | Information on officially-recorded criminal offending was obtained by searching the central computer system of the New Zealand Police, which provides details of all New Zealand convictions and sentences and Australian convictions communicated to the New Zealand Police. Searches were completed following each assessment, at ages 18, 21, 26, 32, and 38 (last search completed in 2013). Official records of criminal conviction were available from 14 years of age onwards, the age from which criminal conviction for all types of offenses was permissible. Criminal offending was coded as a binary variable to reflect whether participants had been convicted. 238/898 (26.5%) of participants had an official record of a criminal offense. |
| **Substance dependence** | Past-year substance dependence was assessed at age 18. Alcohol, cannabis, and hard drug dependence (and use of methadone maintenance) were assessed using DSM-IV criteria. Tobacco dependence was assessed using the FTND. Participants were coded as positive for substance dependence if they met criteria for any disorder or were on methadone maintenance (n=413/1,860 (22.2%)). | Past-year substance dependence was assessed at age 38. Alcohol, cannabis, and hard drug dependence (and use of methadone maintenance) were assessed using DSM-IV criteria. Tobacco dependence was assessed using the FTND. Participants were coded as positive for substance dependence if they met criteria for any disorder or were on methadone maintenance (n=165/886 (18.6%)). |
| **Informant-rated disinhibition** | Informant-report data were collected at age 18. Participants were asked to nominate someone “who knew them well.” These informants were provided with questionnaires and asked to rate each participant on different dimensions. The majority of reports were provided by parents and co-twins.  Informants were asked to rate how much of a problem a series of items were for the participant in the prior 12 months, on a three-point scale (0=not a problem, 1=bit of a problem, 2=yes, a problem). We selected nine items that tapped into disinhibitory problems: “controlling anger, hot temper”; “gets into fights”; “has conflicts with people at work”; “impulsive, rushes into things without thinking what might happen”; “has alcohol problems”; “has cannabis or other drug problems”; “does things against the law”; “lacks self-discipline”; and “makes ‘snap’ decisions (too fast).” Items were summed to create a dimensional scale. Scores were standardized across informants (M=0, SD=1). Informant-reported disinhibition data were available for 1,851 (92.6%) of participants. | Informant-report data were collected at age 38. Participants were asked to nominate someone “who knew them well.” These informants were provided with questionnaires and asked to rate each participant on different dimensions. The majority of reports were provided by friends, partners, and family members.  The same informant-report items were used in the Dunedin Study as in the E-Risk Study. Items were summed to create a dimensional scale. Scores were standardized across informants (M=0, SD=1). Informant-reported disinhibition data were available for 873 (95.1%) of participants. |
| **Lifetime number of sexual partners** | In response to external review, we conducted secondary analyses of risky sexual behavior. At age 18, participants were asked to report the total number of partners with whom they had had sexual intercourse in their lifetime. To reduce the influence of extreme values, we winsorized the variable at the 90th percentile of the distribution (6 partners). | In response to external review, we conducted secondary analyses of risky sexual behavior. At age 38, participants were asked to report the total number of partners with whom they had had sexual intercourse in their lifetime. To reduce the influence of extreme values, we winsorized the variable at the 90th percentile of the distribution (50 partners). |
| ***Family-context correlates*** |  |  |
| **Childhood socioeconomic deprivation** | Childhood socioeconomic status (SES) was defined using a standardized composite of parents' income, education, and social class (Trzesniewski, Moffitt, Caspi, Taylor, & Maughan, 2006). The variable was standardized (M=0, SD=1) and reverse-coded to reflect socioeconomic deprivation. | Childhood SES was measured using a six-point scale that assessed parents’ occupational statuses, defined based on average income and educational levels derived from the New Zealand Census. Parents’ occupational statuses were assessed when participants were born and again at subsequent assessments up to age 15 years. The highest occupational status of either parent was averaged across the childhood assessments (Poulton et al., 2002). The variable was standardized (M=0, SD=1) and reverse-coded to reflect socioeconomic deprivation. |
| **Early maternal**  **age-at-first-birth** | Participants’ mothers were asked to report the age at which they had their first child. The variable was reverse-coded to reflect early age-at-first-birth. The mean maternal age-at-first-birth was 23.5 years (SD=5.9). | Participants’ mothers were asked to report the age at which they had their first child. The variable was reverse-coded to reflect early age-at-first-birth. The mean maternal age-at-first-birth was 22.7 years (SD=3.6). |
| ***Age-at-menarche*** | The mean age-at-menarche reported by female participants was 13.1 years (SD=1.3). | The mean age-at-menarche reported by female participants was 13.0 years (SD=1.1). |

**Statistical analyses**

We used a univariate liability threshold model to estimate genetic, shared environmental, and non-shared environmental influences on reproductive behavior in the E-Risk cohort. We fit a multigroup model with the loadings for genetic and environmental parameters constrained to be the same across men and women. Models were fitted directly to the raw twin data using the method of robust weighted least squares (WLSMV).

We used Poisson regression models with relative risks (RRs)^[[1]](#footnote-1)^ to test whether the polygenic score predicted reproductive behavior and to assess whether childhood socioeconomic deprivation and early maternal age-at-first-birth were correlated with these effects.

We used linear regression to test whether the polygenic score operates through genetic influences on pubertal timing. We tested whether the score predicted female participants’ age-at-menarche.

We used regression to test our central hypothesis that the polygenic score comprises genetic influences on disinhibitory behavior. We analyzed continuously-distributed outcomes (e.g., low childhood self-control) using ordinary least squares. We analyzed binary outcomes (e.g., criminal offending) using Poisson regression models with relative risks.^1^ We analyzed count outcomes (number of sexual partners) using negative binomial regression models with incidence rate ratios (IRRs). We used formal mediation analysis to test whether polygenic-score associations with reproductive behavior were mediated by disinhibition.

In the E-Risk cohort, we corrected all regression and mediation analyses for the non-independence of twin observations by clustering standard errors at the family level. This was accomplished using Taylor series (linearization) variance estimation (for linear regression and mediation analyses) and generalized estimating equations (GEE) for Poisson and negative binomial regression analyses. In the mediation analyses, 95% confidence intervals were obtained from 500 bootstrap replications. Analyses in which men and women were combined were adjusted for sex. Analyses in which E-Risk and Dunedin participants were combined were adjusted for cohort/study. Regression analyses were conducted using SAS version 9.4 (SAS Institute Inc., Cary, NC). Mediation and biometric analyses were conducted using Mplus version 7 (Muthén & Muthén, 1998-2015).

**Appendix S2.** Associations between the age-at-first-birth polygenic score and two disinhibitory outcomes in the Dunedin cohort, using (a) clumping to account for linkage disequilibrium and (b) different *p-*value thresholds for SNP inclusion.

| **Low childhood self-control** | |  |  |  |  |
| --- | --- | --- | --- | --- | --- |
| Non-clumped |  |  |  |  |  |
| PGS | Estimate | SE | t value | p value | adj.R-squared |
| zr_pT_0.0005.x | 0.129 | 0.032 | 4.062 | 5.28E-05 | 0.080 |
| zr_pT_0.001.x | 0.139 | 0.032 | 4.395 | 1.24E-05 | 0.082 |
| zr_pT_0.0015.x | 0.140 | 0.032 | 4.420 | 1.11E-05 | 0.083 |
| zr_pT_0.002.x | 0.144 | 0.032 | 4.563 | 5.74E-06 | 0.084 |
| zr_pT_0.0025.x | 0.147 | 0.032 | 4.649 | 3.83E-06 | 0.085 |
| zr_pT_0.003.x | 0.150 | 0.032 | 4.742 | 2.45E-06 | 0.085 |
| zr_pT_0.0035.x | 0.150 | 0.032 | 4.738 | 2.51E-06 | 0.085 |
| zr_pT_0.004.x | 0.149 | 0.032 | 4.718 | 2.76E-06 | 0.085 |
| zr_pT_0.0045.x | 0.151 | 0.032 | 4.792 | 1.92E-06 | 0.086 |
| zr_pT_0.005.x | 0.152 | 0.032 | 4.796 | 1.89E-06 | 0.086 |
| zr_pT_0.01.x | 0.159 | 0.032 | 5.037 | 5.68E-07 | 0.088 |
| zr_pT_0.015.x | 0.163 | 0.032 | 5.167 | 2.93E-07 | 0.090 |
| zr_pT_0.02.x | 0.163 | 0.032 | 5.173 | 2.83E-07 | 0.090 |
| zr_pT_0.025.x | 0.158 | 0.032 | 4.993 | 7.11E-07 | 0.088 |
| zr_pT_0.03.x | 0.155 | 0.032 | 4.904 | 1.11E-06 | 0.087 |
| zr_pT_0.035.x | 0.150 | 0.032 | 4.745 | 2.42E-06 | 0.085 |
| zr_pT_0.04.x | 0.150 | 0.032 | 4.742 | 2.46E-06 | 0.085 |
| zr_pT_0.045.x | 0.152 | 0.032 | 4.821 | 1.67E-06 | 0.086 |
| zr_pT_0.05.x | 0.150 | 0.032 | 4.758 | 2.28E-06 | 0.086 |
| zr_pT_0.055.x | 0.149 | 0.032 | 4.700 | 3.00E-06 | 0.085 |
| zr_pT_0.1.x | 0.138 | 0.032 | 4.348 | 1.52E-05 | 0.082 |
| zr_pT_0.2.x | 0.125 | 0.032 | 3.939 | 8.82E-05 | 0.079 |
| zr_pT_0.3.x | 0.122 | 0.032 | 3.839 | 1.32E-04 | 0.078 |
| zr_pT_0.4.x | 0.122 | 0.032 | 3.847 | 1.28E-04 | 0.078 |
| zr_pT_0.5.x | 0.121 | 0.032 | 3.803 | 1.53E-04 | 0.078 |
| zr_pT_0.6.x | 0.121 | 0.032 | 3.817 | 1.44E-04 | 0.078 |
| zr_pT_0.7.x | 0.122 | 0.032 | 3.840 | 1.32E-04 | 0.078 |
| zr_pT_0.8.x | 0.122 | 0.032 | 3.843 | 1.30E-04 | 0.078 |
| zr_pT_0.9.x | 0.123 | 0.032 | 3.870 | 1.17E-04 | 0.078 |
| zr_pT_1.x | 0.123 | 0.032 | 3.877 | 1.13E-04 | 0.078 |
|  |  |  |  |  |  |
| Clumped |  |  |  |  |  |
| zr_pT_0.0005.y | 0.091 | 0.032 | 2.874 | 4.15E-03 | 0.071 |
| zr_pT_0.001.y | 0.081 | 0.032 | 2.543 | 1.11E-02 | 0.070 |
| zr_pT_0.0015.y | 0.062 | 0.032 | 1.935 | 5.32E-02 | 0.067 |
| zr_pT_0.002.y | 0.064 | 0.032 | 2.001 | 4.57E-02 | 0.067 |
| zr_pT_0.0025.y | 0.070 | 0.032 | 2.186 | 2.91E-02 | 0.068 |
| zr_pT_0.003.y | 0.081 | 0.032 | 2.526 | 1.17E-02 | 0.069 |
| zr_pT_0.0035.y | 0.087 | 0.032 | 2.729 | 6.47E-03 | 0.071 |
| zr_pT_0.004.y | 0.086 | 0.032 | 2.699 | 7.08E-03 | 0.070 |
| zr_pT_0.0045.y | 0.096 | 0.032 | 3.021 | 2.59E-03 | 0.072 |
| zr_pT_0.005.y | 0.089 | 0.032 | 2.782 | 5.52E-03 | 0.071 |
| zr_pT_0.01.y | 0.110 | 0.032 | 3.447 | 5.92E-04 | 0.075 |
| zr_pT_0.015.y | 0.104 | 0.032 | 3.266 | 1.13E-03 | 0.074 |
| zr_pT_0.02.y | 0.115 | 0.032 | 3.612 | 3.20E-04 | 0.076 |
| zr_pT_0.025.y | 0.104 | 0.032 | 3.273 | 1.10E-03 | 0.074 |
| zr_pT_0.03.y | 0.107 | 0.032 | 3.353 | 8.32E-04 | 0.074 |
| zr_pT_0.035.y | 0.099 | 0.032 | 3.125 | 1.83E-03 | 0.073 |
| zr_pT_0.04.y | 0.107 | 0.032 | 3.372 | 7.77E-04 | 0.074 |
| zr_pT_0.045.y | 0.101 | 0.032 | 3.172 | 1.57E-03 | 0.073 |
| zr_pT_0.05.y | 0.103 | 0.032 | 3.247 | 1.21E-03 | 0.074 |
| zr_pT_0.055.y | 0.101 | 0.032 | 3.183 | 1.51E-03 | 0.073 |
| zr_pT_0.1.y | 0.084 | 0.032 | 2.629 | 8.72E-03 | 0.070 |
| zr_pT_0.2.y | 0.102 | 0.032 | 3.219 | 1.33E-03 | 0.073 |
| zr_pT_0.3.y | 0.102 | 0.032 | 3.209 | 1.38E-03 | 0.073 |
| zr_pT_0.4.y | 0.114 | 0.032 | 3.585 | 3.55E-04 | 0.076 |
| zr_pT_0.5.y | 0.112 | 0.032 | 3.536 | 4.26E-04 | 0.076 |
| zr_pT_0.6.y | 0.114 | 0.032 | 3.589 | 3.50E-04 | 0.076 |
| zr_pT_0.7.y | 0.116 | 0.032 | 3.644 | 2.83E-04 | 0.076 |
| zr_pT_0.8.y | 0.115 | 0.032 | 3.631 | 2.98E-04 | 0.076 |
| zr_pT_0.9.y | 0.114 | 0.032 | 3.595 | 3.41E-04 | 0.076 |
| zr_pT_1.y | 0.114 | 0.032 | 3.591 | 3.47E-04 | 0.076 |

| **Externalizing liability** | |  |  |  |  |
| --- | --- | --- | --- | --- | --- |
| Non-clumped |  |  |  |  |  |
| PGS | Estimate | SE | t value | p value | adj.R-squared |
| zr_pT_0.0005.x | 0.047 | 0.032 | 1.494 | 1.36E-01 | 0.080 |
| zr_pT_0.001.x | 0.059 | 0.032 | 1.863 | 6.28E-02 | 0.082 |
| zr_pT_0.0015.x | 0.062 | 0.032 | 1.964 | 4.98E-02 | 0.082 |
| zr_pT_0.002.x | 0.064 | 0.032 | 2.023 | 4.34E-02 | 0.082 |
| zr_pT_0.0025.x | 0.063 | 0.032 | 2.005 | 4.53E-02 | 0.082 |
| zr_pT_0.003.x | 0.063 | 0.032 | 1.997 | 4.61E-02 | 0.082 |
| zr_pT_0.0035.x | 0.066 | 0.032 | 2.071 | 3.86E-02 | 0.082 |
| zr_pT_0.004.x | 0.064 | 0.032 | 2.030 | 4.26E-02 | 0.082 |
| zr_pT_0.0045.x | 0.066 | 0.032 | 2.088 | 3.71E-02 | 0.083 |
| zr_pT_0.005.x | 0.066 | 0.032 | 2.075 | 3.83E-02 | 0.082 |
| zr_pT_0.01.x | 0.072 | 0.032 | 2.269 | 2.35E-02 | 0.083 |
| zr_pT_0.015.x | 0.081 | 0.032 | 2.563 | 1.05E-02 | 0.085 |
| zr_pT_0.02.x | 0.085 | 0.032 | 2.675 | 7.61E-03 | 0.085 |
| zr_pT_0.025.x | 0.081 | 0.032 | 2.570 | 1.03E-02 | 0.085 |
| zr_pT_0.03.x | 0.079 | 0.032 | 2.502 | 1.25E-02 | 0.084 |
| zr_pT_0.035.x | 0.079 | 0.032 | 2.496 | 1.27E-02 | 0.084 |
| zr_pT_0.04.x | 0.081 | 0.032 | 2.571 | 1.03E-02 | 0.085 |
| zr_pT_0.045.x | 0.085 | 0.032 | 2.692 | 7.23E-03 | 0.085 |
| zr_pT_0.05.x | 0.084 | 0.032 | 2.653 | 8.11E-03 | 0.085 |
| zr_pT_0.055.x | 0.087 | 0.032 | 2.737 | 6.31E-03 | 0.086 |
| zr_pT_0.1.x | 0.083 | 0.032 | 2.637 | 8.51E-03 | 0.085 |
| zr_pT_0.2.x | 0.076 | 0.032 | 2.399 | 1.66E-02 | 0.084 |
| zr_pT_0.3.x | 0.075 | 0.032 | 2.383 | 1.74E-02 | 0.084 |
| zr_pT_0.4.x | 0.076 | 0.032 | 2.414 | 1.60E-02 | 0.084 |
| zr_pT_0.5.x | 0.076 | 0.032 | 2.416 | 1.59E-02 | 0.084 |
| zr_pT_0.6.x | 0.077 | 0.032 | 2.445 | 1.47E-02 | 0.084 |
| zr_pT_0.7.x | 0.078 | 0.032 | 2.479 | 1.34E-02 | 0.084 |
| zr_pT_0.8.x | 0.079 | 0.032 | 2.498 | 1.27E-02 | 0.084 |
| zr_pT_0.9.x | 0.079 | 0.032 | 2.512 | 1.22E-02 | 0.084 |
| zr_pT_1.x | 0.080 | 0.032 | 2.517 | 1.20E-02 | 0.084 |
|  |  |  |  |  |  |
| Clumped |  |  |  |  |  |
| zr_pT_0.0005.y | 0.008 | 0.032 | 0.238 | 8.12E-01 | 0.078 |
| zr_pT_0.001.y | 0.029 | 0.032 | 0.913 | 3.62E-01 | 0.079 |
| zr_pT_0.0015.y | 0.032 | 0.032 | 1.015 | 3.10E-01 | 0.079 |
| zr_pT_0.002.y | 0.022 | 0.032 | 0.694 | 4.88E-01 | 0.079 |
| zr_pT_0.0025.y | 0.031 | 0.032 | 0.961 | 3.37E-01 | 0.079 |
| zr_pT_0.003.y | 0.028 | 0.032 | 0.888 | 3.75E-01 | 0.079 |
| zr_pT_0.0035.y | 0.046 | 0.032 | 1.456 | 1.46E-01 | 0.080 |
| zr_pT_0.004.y | 0.055 | 0.032 | 1.742 | 8.19E-02 | 0.081 |
| zr_pT_0.0045.y | 0.061 | 0.032 | 1.931 | 5.38E-02 | 0.082 |
| zr_pT_0.005.y | 0.056 | 0.032 | 1.768 | 7.74E-02 | 0.081 |
| zr_pT_0.01.y | 0.076 | 0.032 | 2.388 | 1.71E-02 | 0.084 |
| zr_pT_0.015.y | 0.075 | 0.032 | 2.367 | 1.81E-02 | 0.084 |
| zr_pT_0.02.y | 0.083 | 0.032 | 2.635 | 8.56E-03 | 0.085 |
| zr_pT_0.025.y | 0.066 | 0.032 | 2.081 | 3.77E-02 | 0.083 |
| zr_pT_0.03.y | 0.071 | 0.032 | 2.249 | 2.47E-02 | 0.083 |
| zr_pT_0.035.y | 0.069 | 0.032 | 2.180 | 2.95E-02 | 0.083 |
| zr_pT_0.04.y | 0.068 | 0.032 | 2.158 | 3.12E-02 | 0.083 |
| zr_pT_0.045.y | 0.067 | 0.032 | 2.116 | 3.46E-02 | 0.083 |
| zr_pT_0.05.y | 0.054 | 0.032 | 1.695 | 9.03E-02 | 0.081 |
| zr_pT_0.055.y | 0.056 | 0.032 | 1.771 | 7.68E-02 | 0.081 |
| zr_pT_0.1.y | 0.045 | 0.032 | 1.424 | 1.55E-01 | 0.080 |
| zr_pT_0.2.y | 0.043 | 0.032 | 1.344 | 1.79E-01 | 0.080 |
| zr_pT_0.3.y | 0.038 | 0.032 | 1.209 | 2.27E-01 | 0.080 |
| zr_pT_0.4.y | 0.038 | 0.032 | 1.206 | 2.28E-01 | 0.080 |
| zr_pT_0.5.y | 0.035 | 0.032 | 1.100 | 2.72E-01 | 0.079 |
| zr_pT_0.6.y | 0.033 | 0.032 | 1.051 | 2.94E-01 | 0.079 |
| zr_pT_0.7.y | 0.037 | 0.032 | 1.169 | 2.43E-01 | 0.080 |
| zr_pT_0.8.y | 0.036 | 0.032 | 1.137 | 2.56E-01 | 0.079 |
| zr_pT_0.9.y | 0.036 | 0.032 | 1.124 | 2.61E-01 | 0.079 |
| zr_pT_1.y | 0.036 | 0.032 | 1.121 | 2.63E-01 | 0.079 |

**Appendix S3.** Associations between the age-at-first-birth polygenic score and informant-reported Neuroticism.

It is reasonable to ask whether the age-at-first-birth polygenic score predicts risk for psychopathology beyond disinhibitory problems. We therefore investigated whether the polygenic score predicted informant-reported Neuroticism. There was no association between the polygenic score and Neuroticism in either cohort (E-Risk: β = .00, 95% CI [-.04, .05], Dunedin: β = .00, 95% CI [-.06, .07]).

Notes. Informant-reported personality data were collected at age 18 in the E-Risk cohort and age 38 in the Dunedin cohort. Participants were asked to nominate someone “who knew them well.” These informants were provided with questionnaires and asked to describe each participant using a 25-item version of the Big Five Inventory measuring the personality traits of Agreeableness, Conscientiousness, Neuroticism, Extraversion, and Openness to Experience (Benet-Martínez & John, 1998; Richmond-Rakerd et al., 2019). Scores were standardized (M = 0, SD = 1) within the full samples of participants with personality data (E-Risk: N = 2,050, Dunedin: N = 933). Models controlled for sex.

| **Table S1.** Comparison of cross-sex and sex-specific polygenic scores for age-at-first-birth. | | | | | | | | | | | |  |
| --- | --- | --- | --- | --- | --- | --- | --- | --- | --- | --- | --- | --- |
|  | **Males (N = 1,456)** | | | |  | | **Females (N = 1,461)** | | | | |  |
|  |  |  | **Polygenic score association** | | |  | |  |  | **Polygenic score association** | | |
| **Outcome^a^** | **N** | **n (%)** | **Cross-sex score** | **Sex-specific score** | |  | | **N** | **n (%)** | **Cross-sex score** | **Sex-specific score** | |
| Intercourse | 1,343 | 954 (71.0) | 1.03 [1.00, 1.07] | 1.03 [1.00, 1.07] | |  | | 1,393 | 1,028 (73.8) | 1.09 [1.05, 1.13] | 1.08 [1.05, 1.12] | |
| Pregnancy | 1,339 | 100 (7.5) | 1.24 [1.03, 1.50] | 1.11 [0.90, 1.35] | |  | | 1,395 | 148 (10.6) | 1.26 [1.09, 1.46] | 1.24 [1.07, 1.44] | |
| Birth | 1,341 | 24 (1.8) | 1.16 [0.76, 1.79] | 1.37 [0.95, 1.97] | |  | | 1,396 | 52 (3.7) | 1.31 [0.97, 1.75] | 1.35 [1.02, 1.79] | |

Note. Given the limited statistical power for sex-stratified analyses, participants were pooled across cohorts. Effect sizes are risk ratios [and 95% confidence intervals]. Models controlled for cohort/study. The polygenic scores were reverse-coded in analyses, so that higher scores indicate greater genetic risk for early age-at-first-birth.

^a^Binary variables, coded to reflect whether participants had met each reproductive milestone by age-18 or younger.

| **Table S2.** Results from survival models testing polygenic-score associations with continuously-coded reproductive outcomes. | | |
| --- | --- | --- |
| **Outcome** | **N** | **Hazard ratio [95% CI]** |
| Intercourse: E-Risk | 1,825 | 1.17 [1.11, 1.24] |
| Intercourse: Dunedin | 902 | 1.09 [1.02, 1.17] |
| Pregnancy: Dunedin^a^ | 909 | 1.05 [0.98, 1.13] |
| Birth: Dunedin^a^ | 918 | 1.03 [0.96, 1.12] |

Note. Models controlled for sex. The polygenic score was reverse-coded in analyses, so that a higher score indicates greater genetic risk for early age-at-first-birth.

^a^Hazard ratios for age-at-first-pregnancy and age-at-first-birth could not be computed in the E-Risk cohort as there was not sufficient variation in these outcomes at age 18.

| **Table S3.** DZ twin-difference analysis of polygenic-score associations in the E-Risk cohort. | | | | | |
| --- | --- | --- | --- | --- | --- |
|  | |  |  | **Risk ratio [95% CI]** | |
| **Outcome^a^** | **N DZ pairs** | | **n (%)** | **DZ sample** | **Within DZ pairs** |
| Intercourse | 376 | | 511 (68.0) | 1.07 [1.02, 1.12] | 1.03 [0.95, 1.12] |
| Pregnancy^b^ | 371 | | 70 (9.4) | 1.52 [1.21, 1.90] | 1.29 [0.88, 1.88] |
| Birth^b^ | 369 | | 21 (2.8) | 1.61 [1.01, 2.55] | 1.22 [0.57, 2.60] |

Note. Analyses were limited to DZ pairs with complete data on each reproductive outcome. Within-pair associations were estimated from mixed-effects models. Analyses controlled for sex. DZ twins’ polygenic scores were significantly correlated (*r =* 0.54, *p <* .001). Associations within the full E-Risk cohort were as follows: intercourse: RR = 1.08 [1.04, 1.12]; pregnancy: RR = 1.39 [1.19, 1.63]; birth: RR = 1.52 [1.11, 2.07]. The polygenic score was reverse-coded in analyses, so that a higher score indicates greater genetic risk for early age-at-first-birth.

^a^Binary variables, coded to reflect whether participants had met each reproductive milestone by age-18 or younger.

^b^We provide these estimates for full disclosure and so that they can be used in future meta-analytic work. However, they should be interpreted with caution given the relatively small number of DZ twin participants who reported a pregnancy or a birth by age 18.

| **Table S4.** Associations between the age-at-first-birth polygenic score and reproductive and disinhibitory behaviors among women, controlling for age-at-menarche. | | | | | |
| --- | --- | --- | --- | --- | --- |
| **Outcome** | **Effect size** | **N** | **Mean [SD] or**  **n (%)** | **Unadjusted** | **Adjusted for**  **age-at-menarche** |
| *Reproductive behaviors*^a^ |  |  |  |  |  |
| Intercourse | RR | 1,379 | n=1,020 (74.0%) | 1.09 [1.06, 1.13] | 1.09 [1.06, 1.13] |
| Pregnancy | RR | 1,380 | n=147 (10.7) | 1.27 [1.10, 1.47] | 1.25 [1.09, 1.45] |
| Birth | RR | 1,381 | n=51 (3.7%) | 1.34 [1.00, 1.79] | 1.33 [0.99, 1.78] |
| *Disinhibitory behaviors* |  |  |  |  |  |
| Low childhood self-control | β | 1,392 | -0.29 [0.83] | .07 [.01, .13] | .07 [.01, .13] |
| Externalizing liability | β | 1,392 | -0.18 [0.92] | .07 [.02, .13] | .07 [.01, .13] |
| Criminal offending | RR | 1,383 | n=110 (8.0%) | 1.24 [1.01, 1.51] | 1.22 [1.003, 1.50] |
| Substance dependence | RR | 1,384 | n=239 (17.3%) | 1.13 [1.00, 1.28] | 1.13 [1.00, 1.28] |
| Informant-reported disinhibition | β | 1,374 | -0.12 [0.88] | .03 [-.03, .10] | .03 [-.03, .09] |
| Lifetime number of sexual partners | IRR | 1,368 | 6.01 [9.92] | 1.10 [1.04, 1.16] | 1.10 [1.04, 1.16] |

Note. Given the limited statistical power for sex-stratified analyses participants were pooled across cohorts. Models excluded the small number of E-Risk participants who had not started menstruating by the age-18 assessment and the small number of Dunedin participants who were missing data on age-at-menarche. Models controlled for cohort/study. The polygenic score was reverse-coded in analyses, so that a higher score indicates greater genetic risk for early age-at-first-birth. Values in brackets are 95% confidence intervals. β = standardized regression coefficient, RR = risk ratio, IRR = incidence rate ratio.

^a^Binary variables, coded to reflect whether participants had met each reproductive milestone by age-18 or younger.

| **Table S5.** Does childhood disinhibition mediate associations between the polygenic score and reproductive behaviors? | | | | | | | | | |
| --- | --- | --- | --- | --- | --- | --- | --- | --- | --- |
|  |  | | |  | | **Estimate [95% CI]^a^** | | |  |
| **Reproductive behavior^b^** | | **N** | **n (%)** | | **Total effect** | | **Direct effect** | **Total indirect effect** | **% mediation^c^** |
| Intercourse | | 2,736 | 1,982 (72.4) | | .15 [.09, .20] | | .13 [.07, .18] | .02 [.01, .03] | 11% |
| Pregnancy | | 2,734 | 248 (9.1) | | .13 [.07, .21] | | .11 [.05, .18] | .02 [.01, .03] | 16% |
| Birth^d^ | | 2,737 | 76 (2.8) | | .08 [-.04, .18] | | .06 [-.05, .16] | .02 [.01, .03] | 25% |

Note. The polygenic score was reverse-coded in analyses, so that a higher score indicates greater genetic risk for early age-at-first-birth.

^a^Indicates standardized estimates of total, direct, and indirect effects in mediation models.

^b^Binary variables, coded to reflect whether participants had met each reproductive milestone by age-18 or younger.

^c^% mediation was calculated before standardized estimates were rounded to the nearest hundredth.

^d^This analysis should be interpreted with caution as the total effect was not statistically significant.

**Figure S1.** Polygenic prediction effect sizes for the age-at-first-birth score across different cohorts.

Notes. Effect sizes for LifeLines, Swedish Twin Registry, TwinsUK, and Pooled cohorts were taken from Mills et al., 2018. The hazard ratios reported by Mills and colleagues (2018) were inverted to produce a consistent direction of effects with effects from the current analysis, in which the polygenic score was reverse-coded. A hazard ratio for the E-Risk cohort could not be computed as there was not sufficient variation in age-at-first-birth at age 18. Given sample-size constraints, males and females were pooled in the Dunedin cohort. Sample sizes were as follows: LifeLines = 7,154 (females) and 4,611 (males); Swedish Twin Registry = 5,409 (females) and 4,525 (males); TwinsUK = 3,569 (females); Pooled cohorts = 16,132 (females) and 9,136 (males); Dunedin = 918 (full sample).

**Supplementary References**

Abecasis, G.R., Auton, A., Brooks, L.D., DePristo, M.A., Durbin, R.M., Handsaker, R.E., . . . McVean, G.A. (2012). An integrated map of genetic variation from 1,092 human genomes. *Nature*, *491*, 56–65.

Agnew-Blais, J.C., Polanczyk, G.V., Danese, A., Wertz, J., Moffitt, T.E., & Arseneault, L. (2016). Evaluation of the persistence, remission, and emergence of attention-deficit/hyperactivity disorder in young adulthood. *JAMA Psychiatry, 73,* 713–720.

American Psychiatric Association. (1994). *Diagnostic and Statistical Manual of Mental Disorders* (4th ed.). Washington, DC: Author.

Barban, N., Jansen, R., de Vlaming, R., Vaez, A., Mandemakers, J.J., Tropf, F.C., . . . Mills, M.C. (2016). Genome-wide analysis identifies 12 loci influencing human reproductive behavior. *Nature Genetics,* *49,* 1462-1475.

Benet-Martínez, V., & John, O.P. (1998). Los Cinco Grandes across cultures and ethnic groups: multitrait multimethod analyses of the Big Five in Spanish and English. *Journal of Personality and Social Psychology, 75,* 729-750.

Caspi, A., Houts, R.M., Belsky, D. W., Goldman-Mellor, S.J., Harrington, H., Israel, S., ... Moffitt, T.E. (2014). The p factor: One general psychopathology factor in the structure of psychiatric disorders? *Clinical Psychological Science, 2,* 119-137.

Chang, C.C., Chow, C.C., Tellier, L.C., Vattikuti, S., Purcell, S.M., & Lee, J.J. (2015). Second-generation PLINK: Rising to the challenge of larger and richer datasets. *GigaScience*, *4*, Article 7.

Dudbridge, F. (2013). Power and predictive accuracy of polygenic risk scores. *PLOS Genetics*, *9*, Article e1003348.

Euesden, J., Lewis, C. M., & O’Reilly, P. F. (2015). PRSice: Polygenic Risk Score software. *Bioinformatics*, *31*, 1466–1468.

Heatherton, T.F., Kozlowski, L.T., Frecker, R.C., & Fagerström, K.O. (1991). The Fagerström Test for Nicotine Dependence: A revision of the Fagerström Tolerance Questionnaire. *British Journal of Addiction, 86,* 1119-1127.

Howie, B. N., Donnelly, P., & Marchini, J. (2009). A flexible and accurate genotype imputation method for the next generation of genome-wide association studies. *PLOS Genetics*, *5*, Article e1000529.

Mills, M.C., Barban, N., & Tropf, F. (2018). The sociogenomics of polygenic scores of reproductive behavior and their relation to other fertility traits. *RSF: The Russell Sage Foundation Journal of the Social Sciences, 4,* 122-136.

Moffitt, T.E., Arseneault, A., Belsky, D., Dickson, N., Hancox, R.J., Harrington, H.L., ... Caspi, A. (2011). A gradient of childhood self-control predicts health, wealth, and public safety. *PNAS USA 108,* 2693-2698.

Moffitt, T.E., & E-Risk Study Team. (2002). Teen-aged mothers in contemporary Britain. *Journal of Child Psychology* *and Psychiatry*, *43*, 727–742.

Odgers, C.L., Caspi, A., Bates, C.J., Sampson, R.J., & Moffitt, T.E. (2012). Systematic social observation of children’s neighborhoods using Google Street View: A reliable and cost-effective method. *Journal of Child Psychology and* *Psychiatry*, *53*, 1009–1017.

Poulton, R., Caspi, A., Milne, B.J., Thomson, WM., Taylor, A., Sears, M. R., & Moffitt, T.E. (2002). Association between children’s experience of socioeconomic disadvantage and adult health: A life-course study. *The Lancet*, *360*, 1640–1645.

Poulton, R., Moffitt, T.E., & Silva, P.A. (2015). The Dunedin Multidisciplinary Health and Development Study: Overview of the first 40 years, with an eye to the future. *Social Psychiatry & Psychiatric Epidemiology*, *50*, 679–693.

Richmond-Rakerd, L.S., Caspi, A., Arseneault, L., Baldwin, J.R., Danese, A., Houts, R.M., . . . Moffitt, T.E. (2019). Adolescents who self-harm and commit violent crime: testing early-life predictors of dual harm in a longitudinal cohort study. *The American Journal of Psychiatry, 176,* 186-195.

Richmond-Rakerd, L.S., D’Souza, S., Andersen, S.H., Hogan, S., Houts, R.M., Poulton, R., . . . Moffitt, T.E. (in press). Clustering of health, crime and social-welfare inequality in 4 million citizens from 2 nations. *Nature Human Behaviour.*

Robins, L.N., Cottler, L., Bucholz, K.K., & Compton, W. (1995). *Diagnostic interview schedule for DSM-IV*. St. Louis, MO: Washington University School of Medicine.

Schaefer, J.D., Moffitt, T.E., Arseneault, L., Danese, A., Fisher, H.L., Houts, R., ... Caspi, A. (2018). Adolescent victimization and early-adult psychopathology: approaching causal inference using a longitudinal twin study to rule out noncausal explanations. *Clinical Psychological Science, 6,* 352-371.

Sherry, S.T., Ward, M.H., Kholodov, M., Baker, J., Phan, L., Smigielski, E.M., & Sirotkin, K. (2001). dbSNP: The NCBI database of genetic variation. *Nucleic Acids Research*, *29*, 308–311.

Trouton, A., Spinath, F.M., & Plomin, R. (2002). Twins Early Development Study (TEDS): A multivariate, longitudinal genetic investigation of language, cognition and behavior problems in childhood. *Twin Research*, *5*, 444–448.

Trzesniewski, K.H., Moffitt, T.E., Caspi, A., Taylor, A., & Maughan, B. (2006). Revisiting the association between reading achievement and antisocial behavior: New evidence of an environmental explanation from a twin study. *Child Development*, *77*, 72–88.

Ware, E.B., Schmitz, L.L., Faul, J., Gard, A., Mitchell, C., Smith, J.A., . . . Kardia, S.H.R. (2017). Heterogeneity in polygenic scores for common human traits. *bioRxiv.* doi: 10.1101/106062

Wertz, J., Caspi, A., Belsky, D.W., Beckley, A.L., Arseneault, L., Barnes, J.C., ... Moffitt, T.E. (2018). Genetics and crime: integrating new genomic discoveries into psychological research about antisocial behavior. *Psychological Science, 39,* 791-803.

1. Poisson regression models with binary outcomes are the equivalent of negative binomial models. The resulting relative risks are the same. [↑](#footnote-ref-1)
